# A Novel Mapping System for Panoramic Mapping of the Left Atrium: Application to Detect and Characterize Localized Sources Maintaining Atrial Fibrillation

**DOI:** 10.1016/j.jacep.2017.09.177

**Published:** 2018-01

**Authors:** Shohreh Honarbakhsh, Richard J. Schilling, Gurpreet Dhillon, Waqas Ullah, Emily Keating, Rui Providencia, Anthony Chow, Mark J. Earley, Ross J. Hunter

**Affiliations:** Barts Heart Centre, St. Bartholomew’s Hospital, Barts Health NHS Trust, London, United Kingdom

**Keywords:** atrial fibrillation, atrial mapping, catheter ablation, mapping system, rotor, AF, atrial fibrillation, AT, atrial tachycardia, CL, cycle length, LA, left atrium, LAA, left atrial appendage, LVZ, low-voltage zone, PV, pulmonary vein

## Abstract

**Objectives:**

This study sought to use a novel panoramic mapping system (CARTOFINDER) to detect and characterize drivers in persistent atrial fibrillation (AF).

**Background:**

Mechanisms sustaining persistent AF remain uncertain.

**Methods:**

Patients undergoing catheter ablation for persistent AF were included. A 64-pole basket catheter was used to acquire unipolar signals, which were processed by the mapping system to generate wavefront propagation maps. The system was used to identify and characterize potential drivers in AF pre- and post-pulmonary vein (PV) isolation. The effect of ablation on drivers identified post-PV isolation was assessed.

**Results:**

Twenty patients were included in the study with 112 CARTOFINDER maps created. Potential drivers were mapped in 19 of 20 patients with AF (damage to the basket and noise on electrograms was present in 1 patient). Thirty potential drivers were identified all of which were transient but repetitive; 19 were rotational and 11 focal. Twenty-six drivers were ablated with a predefined response in 22 of 26 drivers: AF terminated with 12 and cycle length slowed (≥30 ms) with 10. Drivers with rotational activation were predominantly mapped to sites of low-voltage zones (81.8%). PV isolation had no remarkable impact on the cycle length at the driver sites (138.4 ± 14.3 ms pre-PV isolation vs. 137.2 ± 15.2 ms post-PV isolation) and drivers that had also been identified on pre-PV isolation maps were more commonly associated with AF termination.

**Conclusions:**

Drivers were identified in almost all patients in the form of intermittent but repetitive focal or rotational activation patterns. The mechanistic importance of these phenomena was confirmed by the response to ablation.

## Graphical abstract

**Figure:**
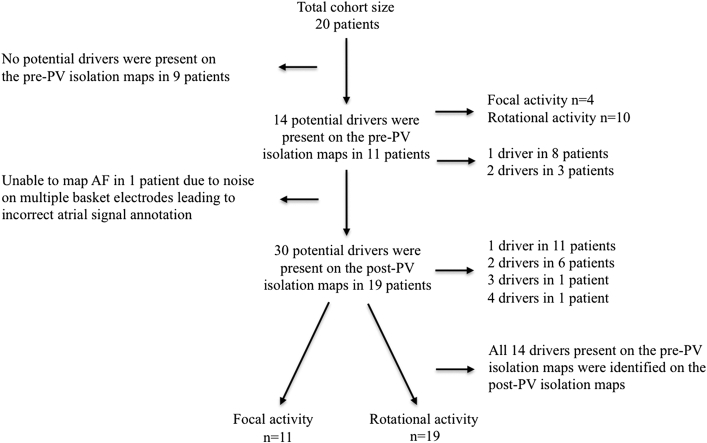

There remains great controversy surrounding the mechanisms sustaining persistent atrial fibrillation (AF). Some groups have demonstrated stable rotors 1, 2 or other spatially conserved drivers, whereas others have not and favor other mechanisms 3, 4. Recent technologies for so called “panoramic atrial mapping” have had some reported success identifying drivers by mapping wavefront activation in AF 1, 5. However, these findings have not been widely replicated as yet. With recent data suggesting that current methods to ablate AF beyond the pulmonary veins (PV) are ineffective (6) there remains great interest in understanding and interrupting the mechanisms sustaining AF.

CARTOFINDER is a novel mapping system that uses the CARTO platform (Biosense Webster, Inc., Baldwin Park, California) as its foundation. It has been developed to map wavefront propagation using multipolar catheters, such as whole-chamber basket catheters, in an open format where location points and electrograms can be scrutinized. Early data using this system have suggested the presence of rotational activity in AF potentially compatible with rotors (7).

This study used the CARTOFINDER system to determine whether there were activation patterns suggestive of localized drivers in AF, in the form of rotational activity or focal activations, which were either stable or recurrent. To distinguish drivers from passive phenomena we hypothesized that they would be unaffected by ablation at remote sites, such as PV isolation, and would bring about a significant response when ablated.

## Methods

Patients undergoing catheter ablation for persistent AF (<24 months and no previous AF ablation) were prospectively included in this study. All procedures were performed with uninterrupted anticoagulation therapy and intravenous heparin administration to achieve an activated clotted time of >300s. The procedures were performed either under conscious sedation or general anesthesia. Patients provided informed consent for their involvement in this study, and the study was approved by the U.K. National Research Ethics Service (16/LO/1379) and complies with the Declaration of Helsinki. It was prospectively registered on clinicaltrials.gov as a substudy of an ongoing trial (NCT02950844).

### Electrophysiology procedure

All cases were performed with CARTO. Right atrial and left atrial geometries were created in all patients. The geometry was acquired using Fast Anatomic Mapping using the PentaRay NAV catheter (Biosense Webster) with an interpolation setting of 17. A high-density bipolar voltage map was created in all patients to establish the relationship between drivers and sites of remodeling. Points were taken using a PentaRay NAV catheter with 2-6-2 mm spacing (Biosense Webster). The interpolation threshold was set to 5 mm for surface color projection and points were collected aiming for complete left atrial coverage (i.e., with no area >5 mm from a data point). Bipolar voltage of <0.5 mV was defined as sites of low-voltage zones (LVZs) (8). To collect the wavefront maps, a 60-mm or 50-mm 64-pole basket catheter was used to record unipolar signals (Constellation, Boston Scientific, Natick, Massachusetts, and FIRMap, Abbott, Menlo Park, California). Both basket catheters consist of 8 splines each with 8 electrodes of 1 mm that are evenly spaced. With the 60-mm Constellation catheter the interelectrode spacing on each spline was 5 mm, whereas with the 60-mm FIRMap catheter this was 9 mm. The catheter was sized from the left atrium (LA) diameters obtained from a transthoracic echocardiogram performed on the day of the procedure as advised by the manufacturer. The basket catheter was positioned in the LA through an 8.5-F catheter SL1 sheath (Daig Medical, Minnetonka, Minnesota) under fluoroscopy guidance. A decapolar catheter (Biosense Webster) was positioned in the coronary sinus. A Thermocool SmartTouch or Thermocool SmartTouch Surround Flow catheter (Biosense Webster) was used for ablation.

Through catheter manipulation the basket catheter was positioned to achieve the best possible atrial coverage. Once in a stable position a recording was taken with CARTOFINDER. A minimum of 2 recordings was taken per patient pre-PV and post-PV isolation. Attempts were made to reposition the basket catheter between recordings to further optimize position, but also just to vary the position of electrodes and orientation of splines slightly. If the operator believed that further catheter positioning could allow for better or different coverage further recordings were taken in a different position. In cases where the coronary sinus activation pattern was predominantly proximal to distal and the shortest LA cycle length (CL) was believed to be at the septum, additional mapping of the right atrium (RA) was permitted following PV isolation.

The CARTOFINDER system provides an evaluation of the coverage achieved as a percentage of the chamber surface area. This allows coverage to be compared between positions and so quantitatively guide catheter repositioning. The CARTOFINDER system calculates coverage through identifying the basket electrodes that are within 10 mm of the geometry. These are then projected onto the geometry and labeled as covering an area with a 10-mm radius. The coverage achieved including these electrodes is then taken as a percentage of the geometry surface area excluding vascular and valvular structures.

### The CARTOFINDER system

The CARTOFINDER system records 30 s of unipolar signals obtained from the 64 poles of the basket catheters by referencing to Wilson’s Central Terminal and filtering between 0.05 and 500 Hz. The signals then undergo processing whereby ventricular far field signals are first filtered. Following this, for each unipolar signal 2 bipolar signals are created by pairing the electrode with the nearest 2 basket electrodes. A bipolar electrogram window is then applied to the unipolar signals that range from earliest onset to the latest offset of the 2 bipolar electrograms. Atrial signals within the bipolar electrogram window are then annotated, whereas areas outside this window are excluded (Online Figure 1). Atrial signals are annotated at the point of the maximum slope (peak negative dv/dt) using wavelet analysis (6). The local activation time is then determined from the annotated unipolar signals. CARTOFINDER creates activation maps during a 250-ms window referencing each electrogram relative to all the others in the LA. This time window then moves through the 30-s recording to show a changing activation map over time.

The available unipolar electrograms can be scrutinized in an open format. The CARTOFINDER system allows manual reannotation of annotated atrial signals by the operator if incorrect annotation has occurred by the system. Following reannotation of the signals the maps are then reprocessed allowing creation of new CARTOFINDER maps with the reannotated signals.

### Mapping in AF

Multiple recordings were taken with the CARTOFINDER system in AF. All maps were assessed for potential drivers by 2 operators during cases and were later scrutinized by 2 blinded observers offline to assess the plausibility of the potential drivers identified. The 2 blinded observers measured the number of consecutive focal or rotational activations at each potential driver site. The number of rotations was assessed visually, rounding down to the nearest 0.5 rotations. During each 30-s recording observers recorded the number of times a potential driver was identified (i.e., the number of times the study definition was met), and the maximum number of cycles during 1 occurrence. For a driver to display temporal periodicity it had to be considered spatially conserved, which we defined as the center of subsequent focal or rotational activations being <1 cm apart.

A potential driver was defined as repetitive patterns of activation that was either focal with radial activation over ≥2 consecutive wavefronts or rotational activity with ≥1.5 rotations of 360° (because these definitions have been used by others previously, NCT02113761). We also assessed: 1) the stability of these phenomena in terms of how many cycles were completed with each occurrence; 2) whether they were repetitive during the recording in which they were observed; and 3) how consistently these phenomena were observed over serial recordings with the basket in different positions. Other wavefront properties were defined along similar lines to previous definitions: planar activation was defined as a single broad wavefront with linear activation, whereas disorganized activity was defined as the absence of clear wavefront propagation (3).

Recordings were taken before and after PV isolation to allow comparison of activation patterns. Recordings taken following PV isolation were used to guide ablation at sites identified as potential drivers. The CL at the driver site was determined manually over the 30-s recording and was the mean interval between atrial complexes on unipolar electrograms recorded on the basket catheter. Where potential drivers were observed both pre- and post-PV isolation, the CL at the driver sites was recorded pre- and post-PV isolation and these measurements were compared.

AF CL was documented pre-PV isolation and monitored following isolation of each PV pair. AF CL was measured over 30 cycles using the PentaRay NAV catheter positioned in the LA appendage (LAA) (9). Following PV isolation a 20-min waiting period was observed before ablation of potential drivers was commenced in the LA body. This was to avoid any delayed effect potentially attributable to PV isolation, which might influence AF CL during ablation at the sites of potential drivers.

To monitor the effect of ablation at the possible driver sites as identified on the post-PV isolation maps, the AF CL was again monitored through the PentaRay NAV catheter positioned in the LAA. Although small changes in CL (usually 5 to 6 ms) have been used previously to determine a response to ablation 10, 11, it was thought that ablation of a clear driver ought to have a more substantial effect. Confirmed drivers were therefore defined as sites where ablation resulted in CL slowing of ≥30 ms, organization of the rhythm to an atrial tachycardia (AT), or termination to sinus rhythm.

### Ablation strategy

PV isolation was achieved with lesions placed 5 to 10 mm outside the venoatrial junction aiming for isolation as ipsilateral PV pairs. The anterior border of the left PVs were ablated on the LAA ridge where possible, or on the appendage side of the ridge where this was unstable. Lesions were delivered on the venous side of the appendage ridge only where this was necessary to isolate PVs.

Ablation at driver sites was delivered with a contact force of 5 to 40 *g*, with a power of 30 to 40 W. Ablation at sites with either focal or rotational activation was delivered as discrete focal points, aiming for the center of the focal or rotational activation. Further ablation was delivered in a cluster of lesions surrounding the first point. Ablation in any region was stopped if the endpoint was met (i.e., AF terminated or CL slowed) or once there was no signal remaining in the area of the focal/rotational activation. The ablation time in any 1 cluster was limited to 5 min. Care was taken not to form a linear lesion with clusters, so as not to impact any AF mechanisms in this way.

Beyond isolating PVs and targeting potential drivers no additional ablation was performed in AF. If the AF organized into an AT this was mapped and ablated.

### Statistical analysis

All statistical analyses were performed using SPSS version 20 (IBM SPSS Statistics, IBM Corp., Armonk, New York). Continuous variables are displayed as mean ± SD or median (interquartile range). Categorical variables are presented as a number and percentage. Kappa was used to examine interobserver agreement between the blinded observers.

## Results

Twenty patients were included in this study. Baseline characteristics are shown in Table 1. All procedures were performed successfully without any complications. The average procedure duration was 250.5 ± 56.5 min and median fluoroscopy time of 1.0 min (interquartile range: 0 to 2 min). The average total ablation time following PV isolation until reaching the study endpoint (AF termination or CL slowing ≥30 ms) was 4.7 ± 1.9 min. The mean LA coverage achieved was 70.3 ± 14.9%. The average number of bipolar voltage points taken was 792 ± 148. The percentage of the LA within 5 mm of a voltage data point was 93.4 ± 5.8%.

**Table 1:** Baseline Characteristics of the Cohort (N = 20)

Age, yrs	61 ± 12
Male	13 (65.0)
Diabetes mellitus	0 (0.0)
Hypertension	7 (35.0)
TIA/CVA	1 (5.0)
Ischemic heart disease	1 (5.0)
Cardiac surgery	1 (5.0)
Left ventricular EF ≥55%	18 (90.0)
LA size, cm^2^	
20–30	17 (85.0)
30–40	3 (15)
>40	0 (0.0)
AF duration, months	13.5 ± 6.2
Previous AT ablation	
Cavotricuspid isthmus-dependent flutter	2 (10.0)
Current antiarrhythmic or rate-controlling strategy	
Beta-blockers including sotalol	11 (55.0)
Amiodarone	5 (25.0)
Flecainide	1 (5.0)
Current anticoagulation strategy	
Warfarin	6 (30.0)
Novel oral anticoagulants	14 (70.0)

Values are mean ± SD or n (%).

AF = atrial fibrillation; AT = atrial tachycardia; CVA = cerebrovascular accident; EF = ejection fraction; LA = left atrium; TIA = transient ischemic attack.

A total of 112 CARTOFINDER maps were constructed in the 20 patients (5.6 ± 1.3 maps per patient). None of the maps required reannotation of the atrial signals. These maps were available to be analyzed within an average of 57 ± 8 s of being collected. Six of these maps were created in the RA (5.4%). The mean time between recordings was 2.9 ± 1.8 min.

**Figure 1:**
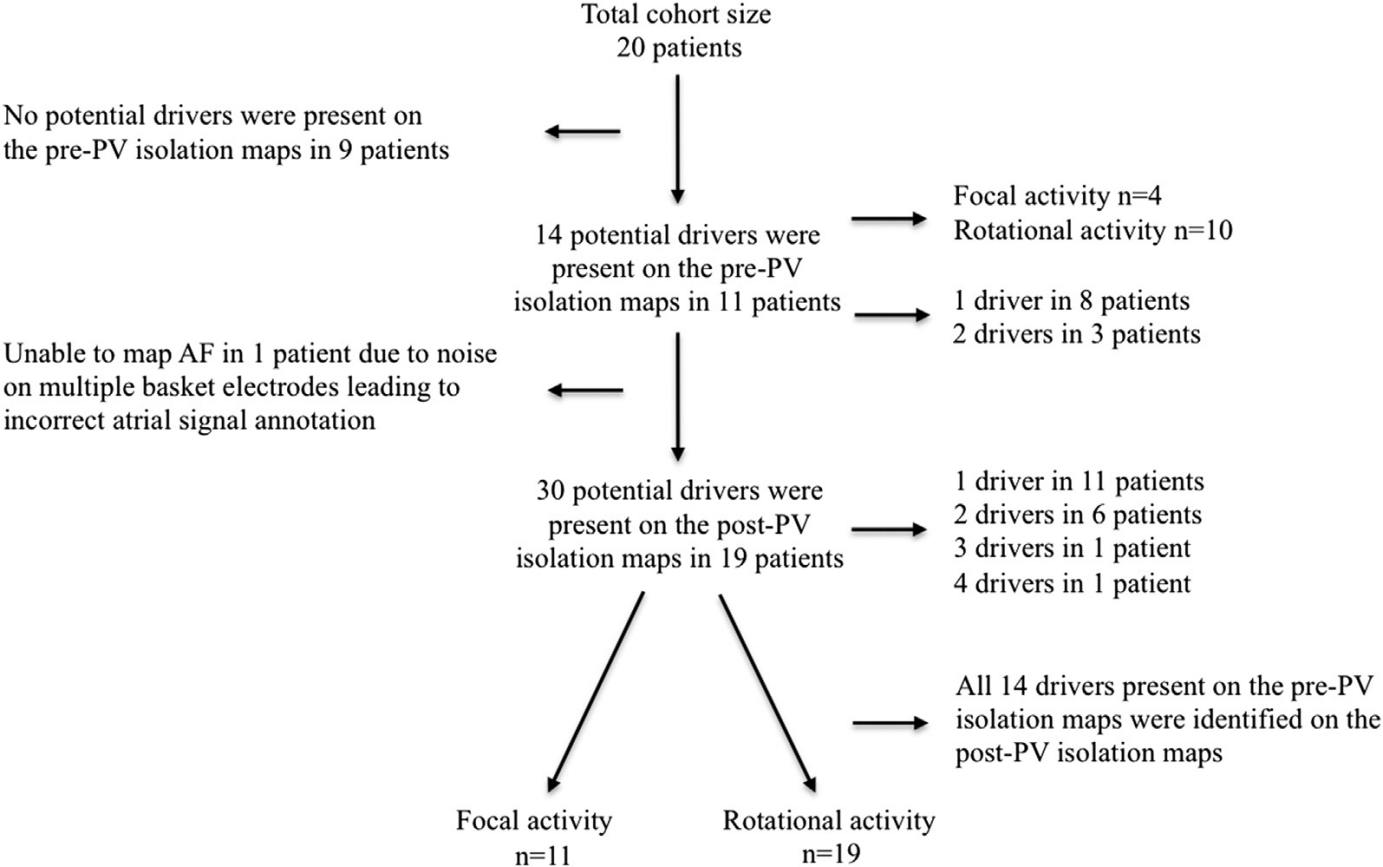
Flow Chart Flow chart that summaries the proportion of drivers that were present on the pre- and post-PV isolation maps and the proportion of these that were of rotational activity and focal activation. AF = atrial fibrillation; PV = pulmonary vein.

Figure 1 shows a flow chart that summaries the proportion of drivers that were present on the pre- and post-PV isolation maps and the proportion of these that were of rotational activity and focal activation.

### AF mapping

#### Pre-PV isolation maps

A total of 59 CARTOFINDER maps were created pre-PV isolation (3.0 ± 0.7 maps per patient). In 11 out of the 20 patients (55.0%) at least 1 driver was identified that met the study definition. The 11 patients had a total of 29 maps out of which 22 maps demonstrated the presence of a driver (75.9%). A total of 14 drivers were identified in the 11 patients (1.3 ± 0.5 drivers per patient, 1 driver in 8 patients, and 2 drivers in 3 patients).

In each of these 11 patients the potential driver was seen in 68.2 ± 16.4% of their pre-PV isolation maps (3 of 14 drivers were present on all maps performed, 2 of 14 in 3 out of 4 maps, 5 of 14 in 2 out of 3 maps, 1 of 14 in 2 out of 4 maps, and 3 of 14 in 1 out of 2 maps). Of the 14 potential drivers, most demonstrated rotational activity (n = 10; 71.4%), with the remainder being of focal activations. In each of the 22 pre-PV isolation maps thought to demonstrate a driver, these were seen to occur 6.5 ± 2.3 times per 30-s recording. The average number of consecutive cycles completed each time the driver definition was fulfilled was 2.5 ± 0.6. The remaining 37 maps pre-PV isolation demonstrated no discernable rotational or focal activity. These maps showed a combination of multiple broad linear wavefront that circulated seemingly randomly, and sites of disorganized activity with no clear discernable wavefront. The mean bipolar voltage (0.35 ± 0.10 mV vs. 0.35 ± 0.18 mV), LA volume (55.4 ± 10.0 ml vs. 57.3 ± 10.3 ml), and duration of AF (13.4 ± 7.1 months vs. 14.1 ± 5.6 months) was similar in those patients in whom drivers were seen on pre-PV isolation maps compared with those where no drivers were seen. However, the mean AF CL pre-PV isolation was longer in those patients in whom drivers were identified on their pre-PV isolation maps suggesting that the AF was more organized (171.8 ± 19.6 ms vs. 141.2 ± 28.0 ms).

#### Post-PV isolation maps

In 1 of the 20 patients with AF the CARTOFINDER maps were generated with inappropriate annotation of atrial signals because of excessive noise on many of the unipolar electrograms presumed caused by damage to the basket. The maps generated in this patient post-PV isolation were therefore limited and did not allow successful driver identification.

Fifty-three CARTOFINDER maps were created post-PV isolation in the remaining 19 patients (2.8 ± 0.8 maps per patient). A total of 30 potential drivers, as per study definition, were identified in these patients with all patients having at least 1 potential driver (range 1 to 4). All 14 of the potential drivers identified pre-PV isolation were also seen on post-PV isolation maps. These drivers were recurrent and occurred on 84.9 ± 20.3% of the CARTOFINDER maps.

### Response to ablation of potential drivers

Of the 30 potential drivers identified, 4 drivers were not targeted because the AF either terminated to AT or sinus rhythm on ablating another driver (n = 2 rotational activity and n = 2 focal activity). The remaining 26 drivers were targeted with ablation out of which 4 potential drivers resulted in no discernible effect (n = 3 rotational activity and n = 1 focal activity; 15.4%). Ablation of the remaining 22 drivers resulted in an effect that confirmed them as a driver as per study definition (84.6% of potential drivers producing a positive response to ablation; 1.2 ± 0.4 per patient). There was at least 1 driver with a positive response to ablation meeting the study definition in all patients.

### Response to ablation of confirmed drivers

**Figure 2:**
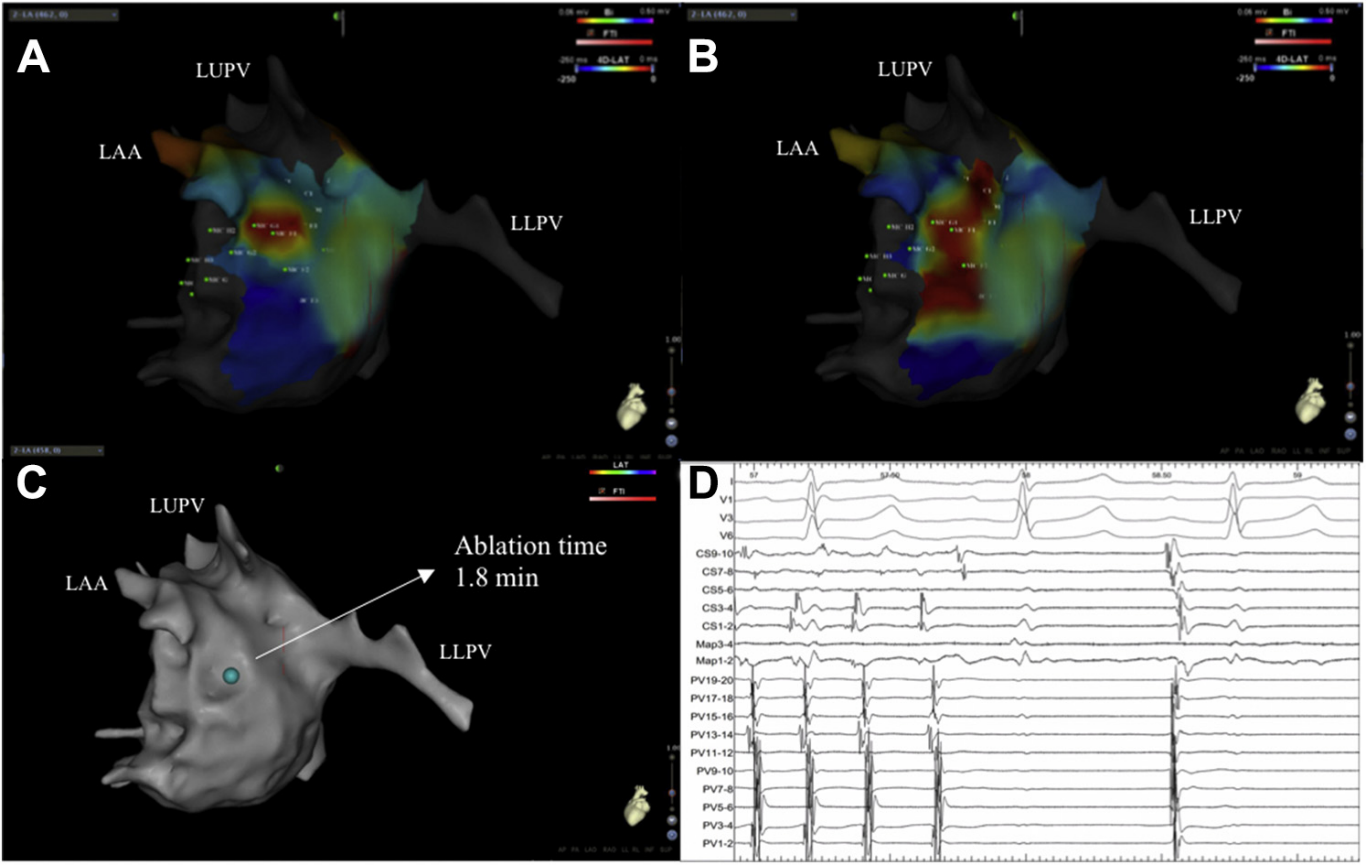
Confirmed Driver With Focal Activation **(A and B)** Still CARTOFINDER maps with an AF driver with focal activity at the lateral wall of the left atrium. **(C)** Site of ablation lesion that achieved sinus rhythm as shown **(D)** on the electrograms including surface electrocardiogram, coronary sinus, and PV signals. LAA = left atrial appendage; LLPV = left lower pulmonary vein; LUPV = left upper pulmonary vein; RUPV = right upper pulmonary vein; other abbreviations as in Figure 1.

Ablation at 12 of the 22 confirmed driver sites resulted in AF termination (Figures 2A to 2D), which accounted for 12 of the 19 patients (63.2%; 5 terminated to sinus rhythm and 7 to AT). The 7 ATs (3 cavo-tricuspid-isthmus dependent flutter, 2 mitral-isthmus dependent flutter, and 2 roof-dependent flutter) were all successfully ablated with termination to sinus rhythm. Ablation at the remaining 10 confirmed driver sites in 7 patients resulted in CL prolongation of ≥30 ms (Table 2). Of the 4 potential drivers that were targeted without any apparent effect this was in patients with >1 potential driver and ablation of other drivers subsequently slowed AF CL ≥30 ms or terminated AF in all patients. Four out of the 8 confirmed focal drivers demonstrated AF termination on ablation, whereas 8 out of the 14 confirmed rotational drivers demonstrated AF termination on ablation (4 of 8, 50% focal drivers vs. 8 of 14, 57.1% rotational drivers). Termination of AF occurred for 10 of the 12 drivers that had been identified on both pre- and post-PV isolation maps, compared with 2 of the 12 drivers that were not identified pre-PV isolation. An average of 3.3 ± 0.9 min of ablation was delivered at each confirmed driver site (3.4 ± 0.9 min for drivers with rotational activity and 3.2 ± 1.0 min for drivers with focal activation).

**Table 2:** Characteristics of the Potential AF Drivers Targeted With the CARTOFINDER System and the Response to Ablation at the Driver Site

Patient ID	Driver Type	Driver Location in LA	Consecutive Repeats in 30 s, Mean ± SD	Proportion of Maps With Driver, %	Driver Visible of Pre-PV Maps	Ablation Response	Ablation Duration, min
#1	Focal	Posterolateral	2.3 ± 0.6	100	Yes	AT	2.8
#2	Rotational Focal	Anterior Lateral	3.3 ± 1.2 4.0 ± 0.8	67 67	No Yes	Nil Sinus	1.8 3.2
#3	Rotational	Posterolateral LAA	2.3 ± 0.6	100	No	SCL	2.4
#4	Focal Focal	Roof/LAA Posterolateral	2.3 ± 0.6 4.0 ± 2.7	50 50	Yes Yes	SCL AT	3.7 4.2
#5	Focal Rotational	Mid anterior Posterolateral	2.3 ± 0.6 2.8 ± 0.4	100 100	No Yes	SCL AT	4.8 3.7
#6	Rotational	Mid anterior	2.2 ± 0.7	75	No	AT	3.3
#7	Rotational	Mid posterior	4.0 ± 0.0	100	No	AT	4.1
#8	Rotational	Inferior RLPV	3.0 ± 0.8	67	Yes	Sinus	3.8
#9	Focal Rotational	Low anterior Mid roof	2.8 ± 1.0 2.9 ± 1.0	100 100	Yes Yes	SCL Sinus	3.5 4.0
#10	Rotational	Posterior LAA	2.3 ± 1.1	75	No	SCL	4.6
#11	Rotational	Anteroseptal	2.5 ± 0.7	100	No	SCL	4.1
#12	Rotational	Mid roof	2.3 ± 0.9	100	Yes	Sinus	3.4
#13	Rotational	Mid anterior	2.4 ± 0.8	100	No	SCL	2.8
#14	Rotational	Anteroseptal	2.2 ± 0.6	100	Yes	AT	3.4
#15	Rotational Focal	Roof/RUPV Roof/LAA	2.5 ± 0.6 2.8 ± 0.8	67 67	Yes Yes	Nil SCL	3.5 2.5
#16	Rotational	Roof	2.3 ± 0.6	100	No	SCL	4.1
#17	Focal	Anterior LAA	3.0 ± 1.3	100	Yes	Sinus	2.2
#18	Rotational	Mid roof	2.8 ± 0.9	67	Yes	AT	2.7
#19	Focal Rotational Rotational	Lateral Anteroseptal Posteroseptal	2.7 ± 0.8 3.3 ± 1.0 3.0 ± 1.3	67 67 50	No No No	Nil Nil SCL	3.5 3.8 2.7

LAA = left atrial appendage; PV = pulmonary vein; RLPV = right lower pulmonary vein; RUPV = right upper pulmonary vein; SCL = slowing cycle length; other abbreviations as in Table 1.

### Comparison of drivers pre- and post-PV isolation

Of the 22 confirmed drivers, 12 had been identified on the pre-PV isolation maps (54.5%) (Table 2). When reviewing the AF CL at the driver sites there was no change in the local CL with PV isolation (138.4 ± 14.3 ms pre-PV isolation vs. 137.2 ± 15.2 ms post-PV isolation). Both pre- and post-PV isolation, the local CL was faster than that recorded at the LAA in all cases. There was no remarkable change in the LAA CL pre- and post-PV isolation (143.5 ± 24.6 ms pre-PV vs. 142.2 ± 25.4 ms post-PV). There was no great difference in number of occurrences seen for a driver pre- and post-PV isolation (6.5 ± 2.3 pre-PV vs. 6.9 ± 2.0 post-PV).

### Relationship of confirmed drivers to LVZs

**Figure 3:**
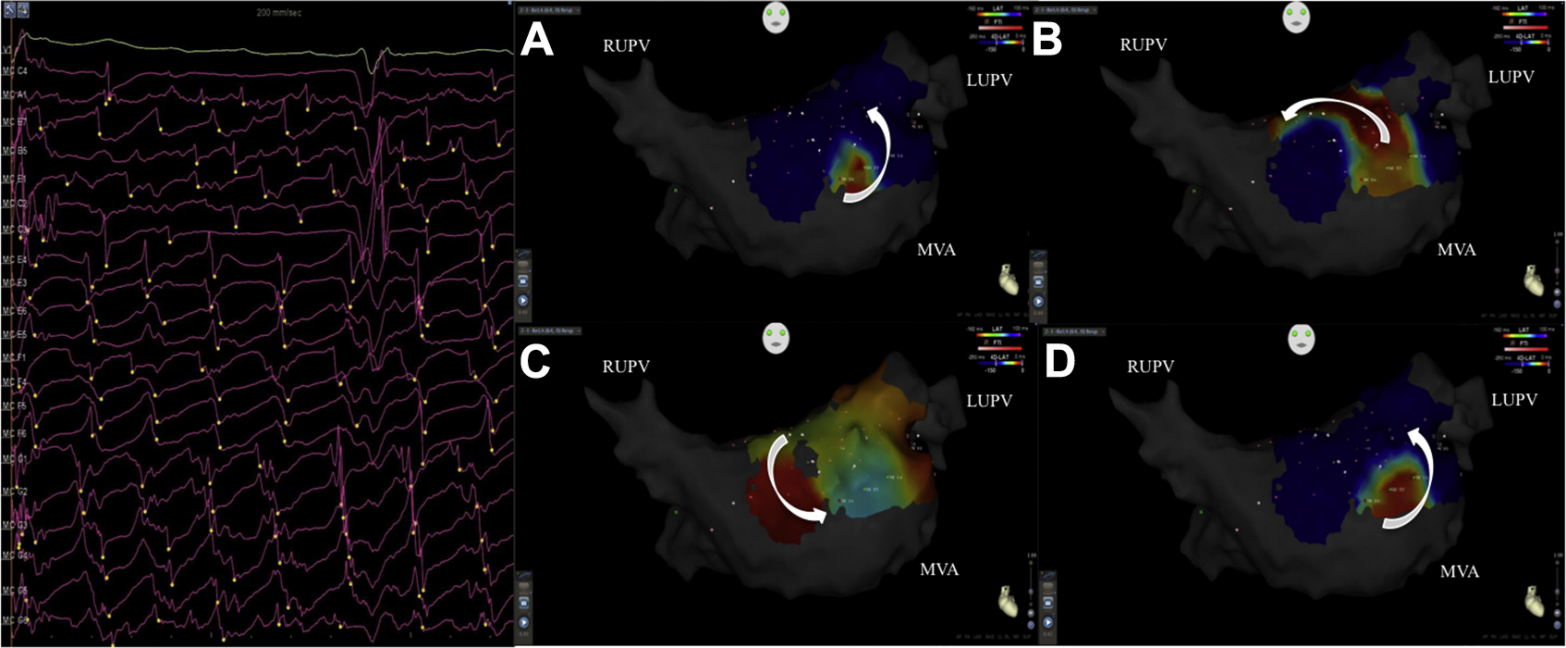
Confirmed Driver With Rotational Activity **(A to D)** Still CARTOFINDER maps demonstrating an AF driver with rotational activity along the anterior wall and the corresponding unipolar electrograms obtained from the basket catheter. This is a good example of real-world unipolar electrograms acquired during AF being annotated by the CARTOFINDER system. It could be debated whether certain deflections should be annotated or not, but with 64 poles this makes up a small proportion of complexes and did not impede generation of maps overall. MVA = mitral valve annulus; other abbreviations as in Figures 1 and 2.

The drivers were predominantly of rotational activity (n = 14; 63.6%) (Figures 3A to 3D), of which 12 occurred at sites of LVZs (85.7%). The remaining 8 drivers were of a focal nature and 4 occurred in areas of normal bipolar voltage (50.0%). The anatomic locations of these drivers are demonstrated in Table 2.

### Temporal stability of drivers

**Figure 4:**
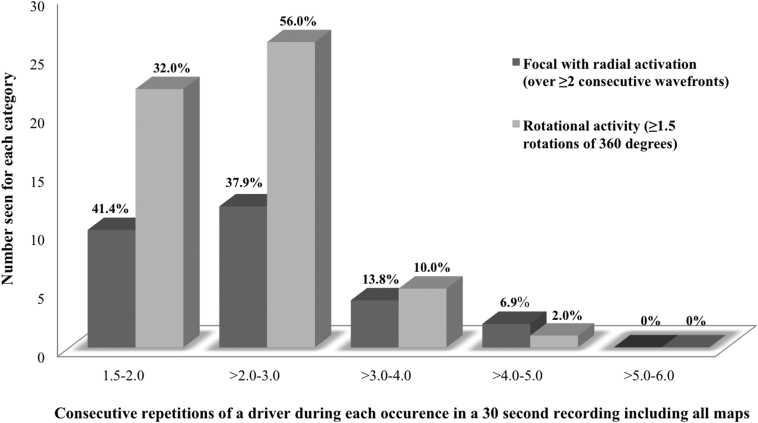
Consecutive Repetitions of a Driver During Each Occurrence Number of times a confirmed AF driver with either rotational **(light grey)** or focal activity **(dark grey)** was seen consecutively in a 30-s recording. Abbreviation as in Figure 1.

The drivers were spatially reproducible and repetitive during a 30-s recording and indeed were seen on separate recordings with different orientations of the basket catheter. Over the 45 recordings of 30 s where potential drivers were confirmed, where rotational activity was observed it occurred 4.4 ± 1.3 times per recording with a mean of 2.6 ± 0.6 consecutive rotations each time. The maximum number of consecutive rotations seen was 4.5. This was reproducible on a per-patient basis: the rotational activity occurred with a mean of 2.7 ± 0.9 consecutive rotations. In those with focal activations, these occurred 4.8 ± 1.9 times per recording with a mean of 2.7 ± 0.3 consecutive focal activations. The maximum number of consecutive focal activations was 5. Only 5.1% of focal and 1.4% of rotational drivers were ever stable for more than 4 consecutive cycles (across all the recordings), and none were seen to complete 6 cycles. These findings were also consistent when reviewing only the 22 confirmed drivers (Figure 4).

### Interoperator variability in the detection of drivers

Examining all AF maps, comparing with the original identification of drivers by the 2 operators performing cases, blinded observer number 1 identified the same drivers in 94% of instances and observer number 2 identified the same drivers in 100%. When examining the potential drivers identified live at the time of the procedure, there was a strong agreement between the 2 blinded observers in terms of whether they identified the same potential drivers or not (Kappa: 0.9; 95% confidence interval: 0.7 to 1.0).

## Discussion

The CARTOFINDER mapping system was effectively used to evaluate the mechanisms of drivers in AF and assess their mechanistic importance through ablation. The drivers identified demonstrated rotational activity in approximately two-thirds of instances and these were mostly confined to LVZs. The remaining one-third were focal activity with radial activation and showed no predilection for LVZs. Nothing compatible with a stable rotor was observed. Drivers were instead transient but recurred at the same locations. The response to ablation at these sites may suggest that these phenomena contribute to maintaining AF. Drivers that had also been identified on pre-PV isolation maps were more commonly associated with AF termination.

### AF drivers and the CARTOFINDER system

Twenty patients had AF mapped with the CARTOFINDER system and 11 of these had potential drivers identified on the pre-PV isolation maps. On the post-PV isolation maps approximately two-thirds of the potential drivers had a rotational activation pattern and one-third were focal. There were no stable drivers and in particular none were compatible with a stable rotor. Instead, drivers were transient, with most lasting ≤4 cycles and none completing 6 cycles. These were repetitive during a 30-s recording and indeed were seen on separate recordings with different orientations of the basket catheter.

There was a response to ablation in all patients. Of the 26 drivers targeted, there was a significant effect with ablation of 22 (12 caused termination of AF and marked CL prolongation occurred for the remainder). The response to ablation may suggest that these drivers participate in the AF process, but further study with long-term outcome data will clarify their importance.

Mechanisms sustaining AF remain controversial. Another group using similar 64-pole basket catheters has reported the presence of continuous stable rotors (1). Others have found little or no evidence of rotors or other spatially conserved drivers 3, 12. Noninvasive mapping with the ECG-I system (CardioInsight Noninvasive 3D Mapping system, Medtronic, Minneapolis, Minnesota) has suggested that AF is maintained by a combination of focal discharges and rotors that, although transient in nature, seem to recur at the same sites (5). The current study is most compatible with the findings of this latter study.

Although the re-entry mechanisms observed were compatible with rotors, with the limited mapping resolution and the transient nature of the phenomena it was difficult to be sure of this from the maps obtained. Drivers with rotational activation were predominantly located in LVZs suggesting that they may be anchored to areas of structural remodeling or scar. It is also recognized that the focal discharges seen on endocardial mapping may not have been truly focal and could well represent endocardial breakthrough because of dissociation of the endocardial and epicardial layers (13). There was no predilection of drivers for any particular LA anatomic site. Drivers were identified bordering the LAA in 5 patients (because the LAA itself is not mapped). These were ablated at the LAA border and no patients required ablation within the LAA as such.

Daoud et al. (7) reported on the mapping of AF with the CARTOFINDER system. In contrast to their findings, we were able to identify drivers on CARTOFINDER maps created pre-PV isolation in 58% of patients compared with their 86% of patients. In the current study drivers were identified pre-PV isolation more often in those with more organized AF as evidenced by a longer AF CL at the start. In the remaining patients a combination of broad planar wavefronts and disorganized, chaotic activity was seen. Drivers were demonstrated in a much greater proportion of patients post-PV isolation. The 2 potential explanations for this are either that we failed to detect drivers pre-PV isolation, or that there were genuinely more drivers present post-PV isolation. Several studies have shown an organizing effect of PV isolation 10, 14. It is possible that PV isolation may eliminate areas of wavebreak and organizes wavefronts to form re-entry (15). It is also possible, however, that other drivers were present pre-PV isolation at the conical PV ostia that were not mapped with a spherical basket in the body of the LA. Alternatively, limitations of this mapping technique may not have allowed identification of drivers in rapid disorganized AF.

Notably, targeting the drivers identified on both pre- and post-PV isolation maps more frequently resulted in termination of AF, suggesting that greater stability might mean greater mechanistic importance. Furthermore, the CL of these driver sites was not influenced by PV isolation, which highlights their independent role as AF drivers.

Although there are other panoramic mapping systems, the CARTOFINDER system is novel in that it creates global activation propagation maps using local activation times obtained through annotation of atrial signals. This differs from phase mapping used by the TOPERA (Abbott, Menlo Park, California) or ECG-I systems, which assumes the presence of cyclical phenomena, such as rotors, and seeks to demonstrate these. CARTOFINDER and TOPERA both rely on the use of whole-chamber basket catheters. However, the TOPERA system relies on 2-dimensional maps and assumes the basket catheter splines to be stationary and undistorted, whereas CARTOFINDER provides dynamic 3-dimensional anatomic location data for the basket catheter and displays activation maps on the left atrial geometry to facilitate targeting of ablation.

### Study limitations

We intentionally used a broad definition of potential drivers so as to maximize sensitivity, but recognize that even this may have missed important phenomena, such as intramural re-entry. Where drivers were not identified it is unclear whether this is because drivers were outside the area mapped, were mapped but not detected, or were not present.

To determine the mechanistic significance of potential drivers we necessarily focused on electrophysiological endpoints because there is arguably no other way to verify that a driver has been mapped. Although termination of AF is clear, the importance of CL prolongation is less certain. Others have used termination of AF or CL prolongation as a surrogate for the interruption of mechanisms important for the maintenance of AF 1, 10, 11, 16 (NCT02113761). In a patient who may have multiple potential drivers it would seem important to note any marked response to ablation. In defining a significant change in AF CL as ≥30 ms we have used the most stringent criteria of any published study reporting AF CL. Nevertheless, even if we only confirmed as drivers those phenomena where AF terminated altogether, the results of this study would be very similar albeit with fewer confirmed drivers. Although the response to ablation suggests that these drivers may participate in the mechanisms sustaining AF, further studies powered to evaluate long-term outcome are required to determine the clinical effectiveness of targeting these phenomena.

Although drivers displayed spatial stability these data suggest temporal periodicity. However, it is possible that this represents intermittent failure to detect drivers perhaps because of electrogram fractionation (17).

## Conclusions

This novel mapping system was effectively used to map wavefront activation in AF. Nothing compatible with a stable rotor was identified, but transient focal and rotational sources were identified that recurred frequently at the same sites. Drivers with a rotational activation pattern were mostly confined to LVZs, whereas focal activations were not. The response to ablation may suggest that these drivers participate in the AF process. However, further characterization of these phenomena remains desirable and randomized studies with long-term follow-up are needed to determine the clinical impact of their ablation.

Perspectives **COMPETENCY IN MEDICAL KNOWLEDGE:** The success rates for catheter ablation of persistent AF are limited. Uncertainty remains as to the mechanisms sustaining persistent AF. Targeting of drivers has been reported albeit with variable success and this remains controversial. The system was then used to identify and characterize drivers in AF. These were focal or rotational activity, which were intermittent but recurred at the same sites repeatedly. Rotational activity often occurred in areas of low voltage suggesting structural remodeling. Targeting these drivers terminated AF in two-thirds of patients and slowed CL substantially in the remainder.

**TRANSLATIONAL OUTLOOK:** This study demonstrated clinical utility of the CARTOFINDER mapping system in targeting AF drivers. The endpoints were therefore electrophysiological. Randomized controlled studies with long-term follow-up are needed to determine whether targeting these impacts outcomes. The ideal way to identify drivers also remains to be seen and further comparisons of the findings with the currently available technologies remains desirable.
