# Supplementary material for: A Novel Mapping System for Panoramic Mapping of the Left Atrium: Application to Detect and Characterize Localized Sources Maintaining Atrial Fibrillation
Source: JACC Clin Electrophysiol. 2018 Jan;4(1):124–34. doi: 10.1016/j.jacep.2017.09.177 (PMC5777816; doi:10.1016/j.jacep.2017.09.177)
Supplement: Online Figure 1 [file mmc1.docx]

Online Appendix


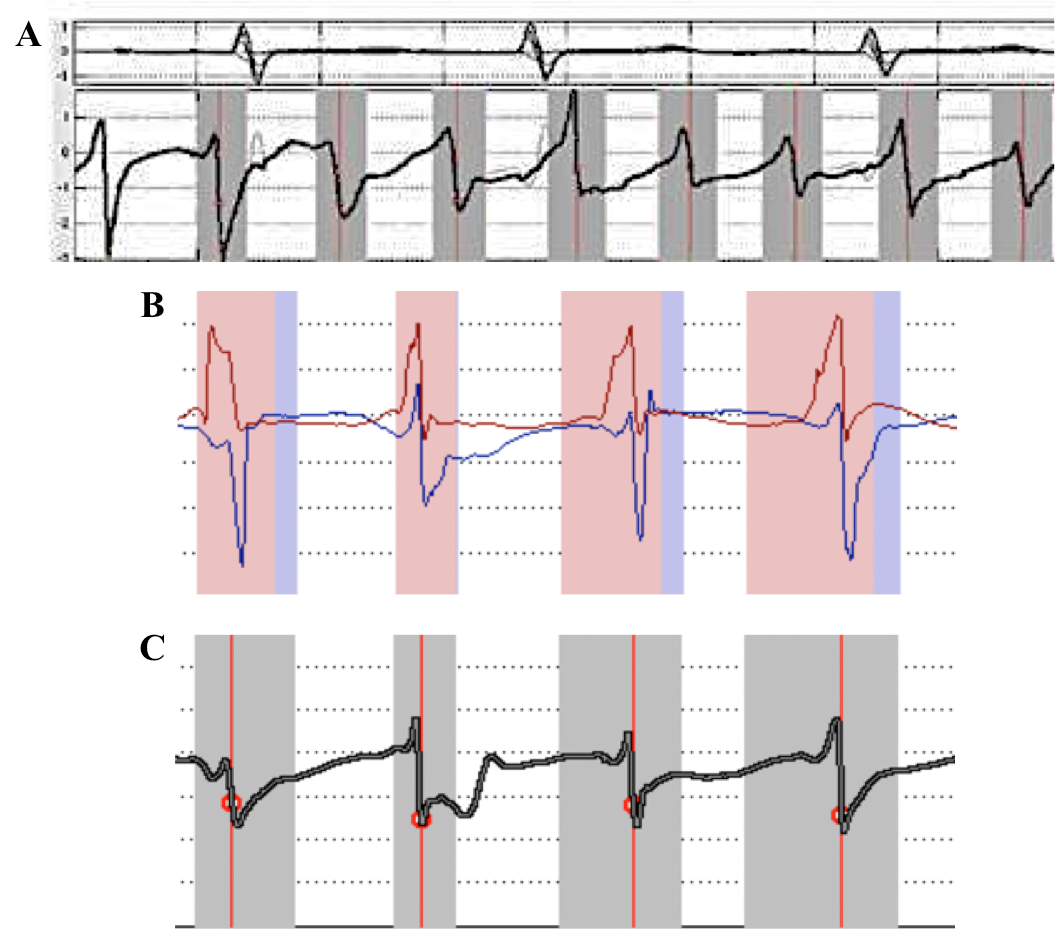


**Figure 1A-C**

**(A)** Demonstrates the pure atrial unipolar signals following far field ventricular signal filtering. The faint grey line demonstrates the initial signal prior to filtering.

**(B)** Shows the bipolar electrogram window made up of two bipolar electrograms created by pairing the electrode with the nearest two basket electrodes. Red and blue electrograms represents each bipole and the bipolar electrogram window ranges from the earliest onset to the latest offset of the two bipolar electrograms.

(**C)**Atrial signals that are within the bipolar electrogram window are then annotated and through wavelet analysis sites of earliest activation in relation to the other electrodes are identified.
